# Antimicrobial Activity of Some Novel Armed Thiophene Derivatives and Petra/Osiris/Molinspiration (POM) Analyses

**DOI:** 10.3390/molecules21020222

**Published:** 2016-02-17

**Authors:** Yahia Nasser Mabkhot, Fatima Alatibi, Nahed Nasser E. El-Sayed, Salim Al-Showiman, Nabila Abdelshafy Kheder, Abdul Wadood, Abdur Rauf, Saud Bawazeer, Taibi Ben Hadda

**Affiliations:** 1 Department of Chemistry, College of Science, King Saud University, P. O. Box 2455, Riyadh-11451, Saudi Arabia; fatmah-alotaibi@hotmail.com (F.A.); nelsayed@ksu.edu.sa (N.N.E.-S.); showiman@ksu.edu.sa (S.A.-S.); 2 National Organization for Drug Control and Research, Agouza, Giza 35521, Egypt; 3 Department of Pharmaceutical Chemistry, Faculty of Pharmacy, King Khalid University, Abha 61441, Saudi Arabia; nabila.abdelshafy@gmail.com; 4 Department of Chemistry, Faculty of Science, Cairo University, Giza 12613, Egypt; 5 Department of Biochemistry, UCSS, Abdul Wali Khan University Mardan, Mardan 23250, Pakistan; awadood2001@yahoo.com; 6 Department of Geology, University of Swabi, Khyber Pakhtunkhwa, Anbar 23561, Pakistan; mashaljcs@yahoo.com; 7 Department of Pharmaceutical Chemistry, Faculty of Pharmacy, Umm Al-Qura University, P. O. Box 42, Makkah 21955, Saudi Arabia; saud.bawazeer@outlook.com; 8 LCM Laboratory, Faculty of Sciences, University of Mohamed Premier, Oujda 60000, Morocco; taibi.ben.hadda@gmail.com

**Keywords:** armed thiophenes, antimicrobial activity, Petra/Osiris/Molinspiration (POM) analyses

## Abstract

Tetrasubstituted 2-acetylthiophene derivative **5** was synthesized and then condensed with various nitrogen nucleophiles such as 5-amino-1,2,4-triazole, 2-aminobenzimidazole, aniline or *p-*chloroaniline to afford the corresponding iminothiophene derivatives **6**–**8a**,**b**. Condensation of thiophene **5** with malononitrile as carbon nucleophile afforded compound **9**, which underwent nucleophilic addition with DMF-DMA to afford compound **10**. The newly synthesized products were characterized by elemental analysis, IR, MS, ^1^H-^13^C-NMR and CHN analysis and then evaluated for their antimicrobial activity. Results of the *in vitro* antibacterial activity showed that thiophene derivative **7** was found to be more potent than the standard drug gentamicin against *Pseudomonas aeruginosa*. Some of these compounds showed potential antimicrobial activities. Molecular docking and Osiris/Molinspiration analyses show the crucial role and impact of substituents on bioactivity and indicate the unfavorable structural parameters in actual drug design: more substitution with electronic donor group doesn’t guarantee more effective bioactivity. This study should greatly help in an intelligent and a controlled pharmacomodulation of antibiotics.

## 1. Introduction

**Figure 1:**
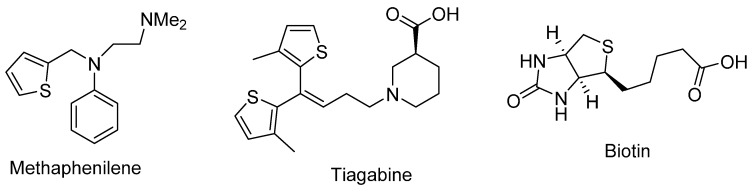
Example of important bioactive thiophenes.

The thiophene ring is present in numerous pharmacologically important compounds and natural products [1,2,3,4,5,6,7]. Highly substituted thiophenes have been synthesized by various methodologies and examined for diverse pharmacological activities [8,9,10,11,12]. These include the antiallergic agent methaphenilene, the anticonvulsant tiagabine, and biotin which is used for preventing and treating biotin deficiency associated with pregnancy (Figure 1).

1,2,4-Triazoles are useful key subunits which found applications in diverse therapeutic fields as antibacterial [13,14], antifungal [15], analgesic [16], anti-inflammatory [17] and anticancer agents [18]. In addition, benzimidazoles are important structural motifs of a variety of biologically active compounds with antibacterial [19], antiviral [20], anticancer [21,22], antifungal [23], and antioxidant properties [24].

Prompted by these findings and as a continuation of our research program on the chemistry of thiophene derivatives [25,26,27,28], we report herein the synthesis using simple experimental procedures of some novel thiophene derivatives obtained using the readily accessible 2-acetylthiophene derivative **5** and incorporating triazole, benzimidazole, and substituted aniline moieties. The antimicrobial activities of the synthesized compound were also investigated. A comparison between experiment and theoretical predictions of the antibacterial activity has enabled us to identify alternative combined pharmacophore sites structures. The nature of pharmacophore site assignment of the thiophene compounds was based on their docking and Petra/Osiris/Molinspiration (POM) analyses.

## 2. Results and Discussion

### 2.1. Synthesis

**Scheme 1:**
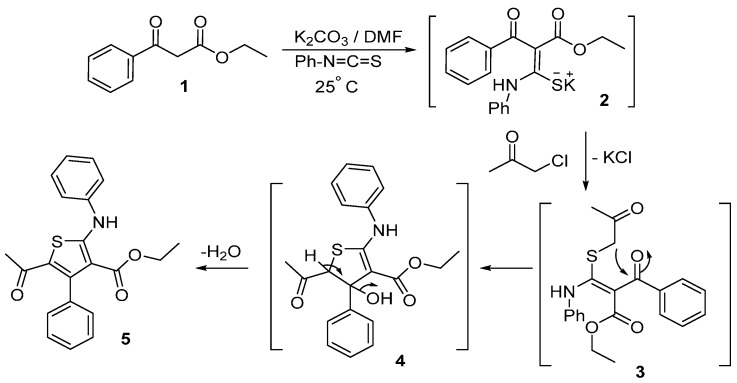
Synthesis of ethyl 5-acetyl-4-phenyl-2-(phenylamino)thiophene-3-carboxylate (**5**).

Preparation of the target compound **5** started with the reaction of ethyl benzoylacetate (**1**) with phenyl isothiocyanate in the presence of K_2_CO_3_ and DMF as a solvent to afford the intermediate ketene *N*,*S*-acetal salt **2**. Treatment of the potassium salt **2** with chloroacetone gave the desired ethyl 5-acetyl-4-phenyl-2-(phenylamino)thiophene-3-carboxylate (**5**) in good yield (Scheme 1).

The reaction proceeds via nucleophilic displacement of chloride to give *S*-alkylated intermediate **3**, followed by nucleophilic addition of the anion of the CH**_2_** group to the carbonyl group of the benzoyl moiety to give the intermediate **4**. The latter is converted into the thiophene derivative **5** via loss of a water molecule (Scheme 1).

The structure of tetrasubstitutedthiophene **5** was established on the basis of its elemental analyses and spectral data. The IR spectrum of compound **5** showed absorption bands at 3229, 1656, and 1623 cm^−1^ due to NH and two carbonyl functions. The ^1^H-NMR spectrum of **5** showed a triplet signal at δ 0.66 (*J* = 7.1 Hz) due to CH_3_ protons, a quartet signal at δ 3.84 (*J* = 7.1 Hz) due to CH_2_ protons. The spectrum showed also other proton signals at δ 1.67 and 10.96 (D_2_O-exchangeable) ppm for CH_3_ and NH, respectively, in addition to aromatic multiplets at δ 7.10–7.40 ppm. Also, its ^13^C-NMR spectral data also confirmed the assigned structure and revealed the presence of 17 signals as expected.

**Scheme 2:**
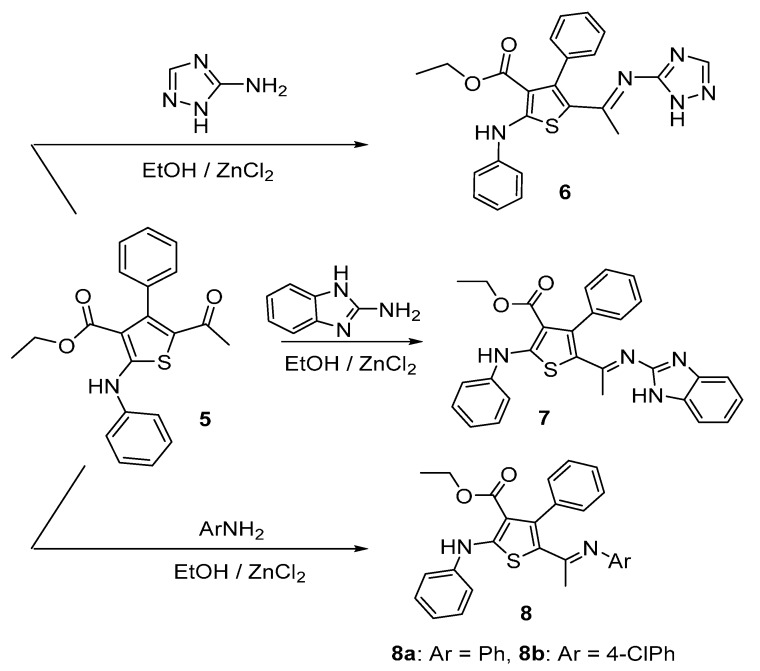
Condensation of acetyl thiophene **5** with various nitrogen nucleophiles.

The reactivity of acetylthiophene **5** towards various nitrogen nucleophiles was then investigated as depicted in Scheme 2. Accordingly, treatment of acetylthiophene **5** with 5-amino-1,2,4-triazole, 2-aminobenzoimidazole, aniline or 4-chloroaniline under reflux in the presence of TEA, ZnCl_2_ and EtOH as solvent furnished in each case, one isolable product (as verified by TLC). The reaction products were identified as iminothiophene derivatives **6**–**8a**,**b** (Scheme 2). Formation of these imino derivatives was confirmed based on their IR, ^1^H-NMR and ^13^C-NMR spectral data.

**Scheme 3:**
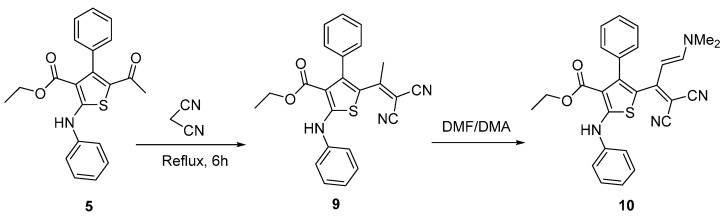
Synthesis of thiophene derivatives **9** and **10**.

Knoevengel condensation of thiophene **5** with malononitrile gave thiophene derivative **9** (Scheme 3). Heating thiophene **9** with DMF/DMA afforded enaminone **10**. The structures of thiophene derivatives **9** and **10** were confirmed using their IR, ^1^H-NMR and MS. The IR spectra of products **6**–**8a**,**b** revealed absence of absorption bands due to the ketone carbonyl group of the starting material and the appearance of new absorption bands due to the C=N group in the 1590–1625 cm^−1^ region. ^1^H-NMR spectrum of imino derivative **6** or **7** showed two D_2_O exchangeable signals due to two NH protons. Furthermore, mass spectra for compound**s** **6** and **7** showed the molecular ion peak at *m*/*z* 431 and at *m*/*z* 480 (4.9%), respectively.

The ^1^H-NMR spectrum of thiophene **10** revealed the presence of the two methyl groups of the dimethylamino moiety, as indicated by the presence of two singlet signals at δ 3.3 and 3.1 ppm. In addition to this, it revealed the presence of the characteristic two signals for the olefinic protons at δ 6.9 and 5.3 (*J* = 12 Hz).

### 2.2. Antimicrobial Evaluation

We investigated *in vitro* antibacterial activity of the newly synthesized compounds against two Gram-positive and two Gram-negative bacteria known to cause infections in humans. The antifungal activity of these compounds was also evaluated against four fungal species. Diameter of the inhibition zone was used as a criterion for the antimicrobial activity using a well diffusion agar method. We also included standard antimicrobial agents in the same assay to compare the potency of the tested compounds. The results are depicted in Table 1 and Table 2.

**Figure 2:**
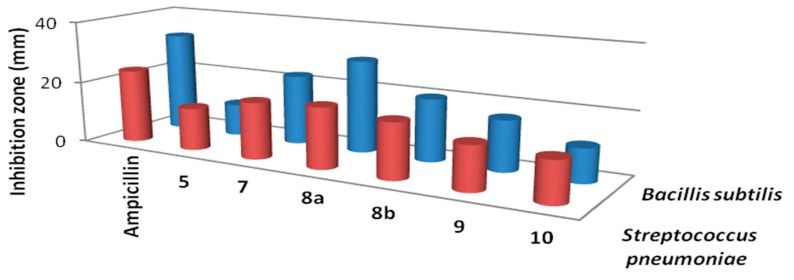
Activity of the synthesized thiophene derivativesagainstGram positive bacteria.

**Figure 3:**
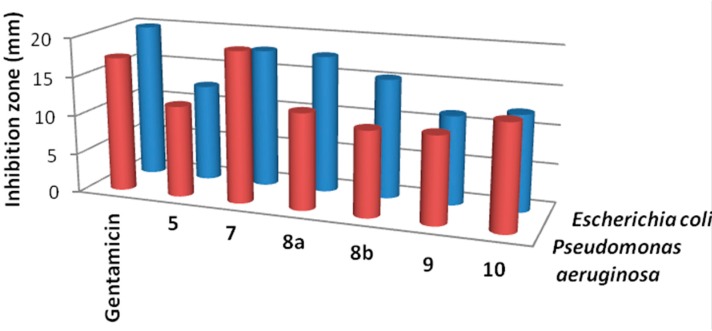
Activity of the synthesized thiophene derivatives against Gram negative bacteria.

**Figure 4:**
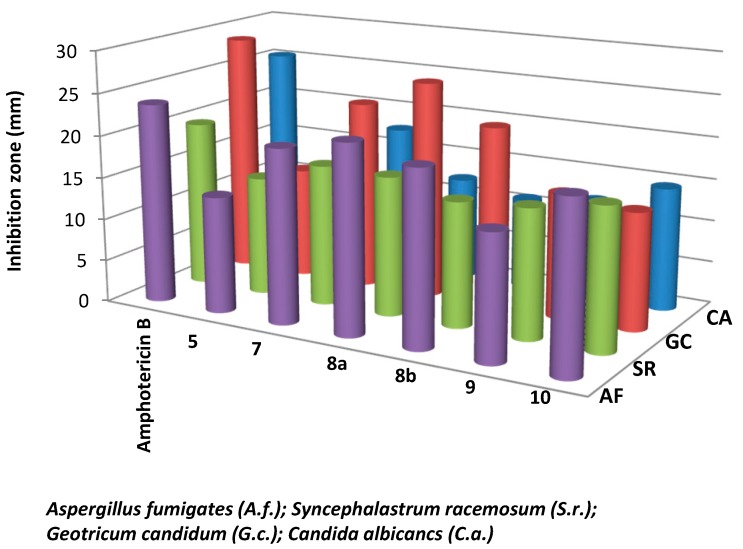
Antifungal activity of the synthesized thiophene derivatives.

Results of the *in vitro* antibacterial activity revealed that compounds **7**, **8a** and **8b** had the best antibacterial activity against all tested bacteria (Table 1, Figure 2 and Figure 3). It is widely known that Gram-negative bacteria are more resistant to antimicrobial agents than are Gram-positive ones [29]. This intrinsic resistance is attributed to the lipopolysaccharide outer membrane which acts as an efficient permeability barrier [30,31]. This, on the other hands, makes the results of the synthesized thiophene derivatives against *Pseudomonas aeruginosa* very promising. Also, the results in Table 1 revealed that thiophene derivative **7** was found to be more potent than the standard drug gentamicin against *Pseudomonas aeruginosa*. Data from the antifungal evaluation have shown that compounds **7**, **8a**, **8b** and **10** were also active against the four tested fungal species (Figure 4, Table 2). The results suggest that the new skeletons possessing benzimidazole and thiophene moieties may provide valuable leads for the synthesis and development of novel antimicrobial agents.

### 2.3. Molecular Docking

Molecular docking is used to predict the binding mode of ligands within the binding site of target proteins [32]. To validate and specify the target protein for the antifungal and antibacterial activity of the newly synthesized tetrasubstituted thiophene derivatives, like in a previous study, nine different target proteins *i.e.*, dihydrofolate reductase (DHFR) (PDB ID 4HOF), secreted aspartic protease (PDB ID 3Q70) and N-myristoyl transferase (PDB ID 1IYL) from *Candida albicans* were selected as antifungal targets, whereas for anti-bacterial targets dihydrofolate reductase (PDB ID 3FYV), gyrase B (PDB ID 4URM) and sortase A (PDB ID 2MLM) from *Staphyllococcus aureus* and rhomboid protease (PDB ID 3ZMI) from *Escherichia coli* were downloaded from the Protein Data Bank. All the tetrasubstituted thiophene derivatives were docked into the active site of these nine selected target proteins. After analysis of the docking results it was found that these compounds showed good interactions with two target proteins, *i.e.*, dihydrofolate reductase from *C. albicans* and rhomboid protease from *E. coli*, while showing very poor interaction or no interactions with the rest of the target proteins. Therefore, the binding mode of these compounds within the active sites of dihyrofolate reductase and rhomboid protease were studied in more detail.

**Figure 5:**
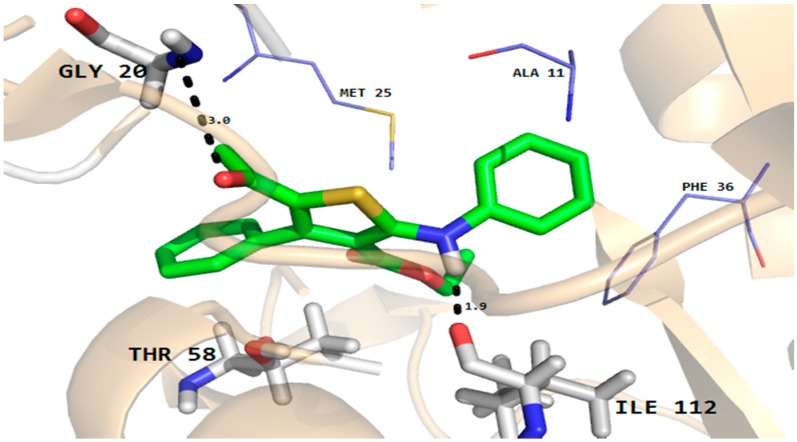
Molecular docking conformation of compound **5** in the active site of *C. albicans* dihydrofolate reductase protein (PDB ID 4HOF) showing hydrogen bonding, hydrophobic and van der Waals interactions.

The docking results showed that all the compounds fit well in the binding pockets of dihydrofolate reductase and rhomboid protease target proteins. In the case of compound **5**, good interactions with the dihydrofolate reductase and rhomboid protease were observed. As shown in Figure 5, compound **5** established two hydrogen bonds with important active site residues (Gly20 and Ile112) and a number of hydrophobic and van der Waals (VDW) interactions with active site residues (Ala11, Met25 and Phe36 *etc*.) of dihydrofolate reducatase.

**Figure 6:**
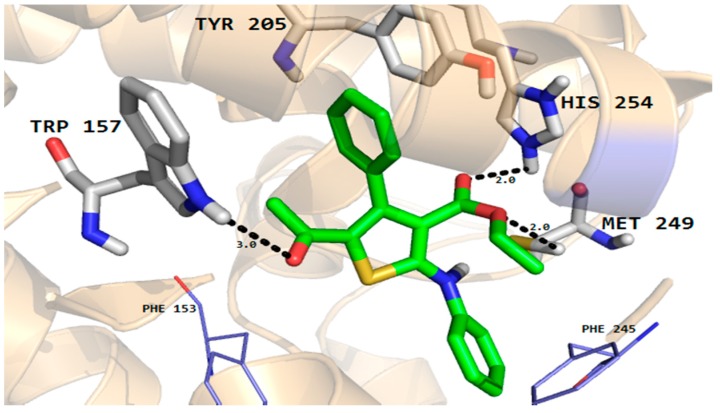
Molecular docking conformation of compound **5** in the active site of rhomboid protease of *E. coli* (PDB ID 3ZMI) showing hydrogen bonding, hydrophobic and van der Waals interactions.

**Figure 7:**
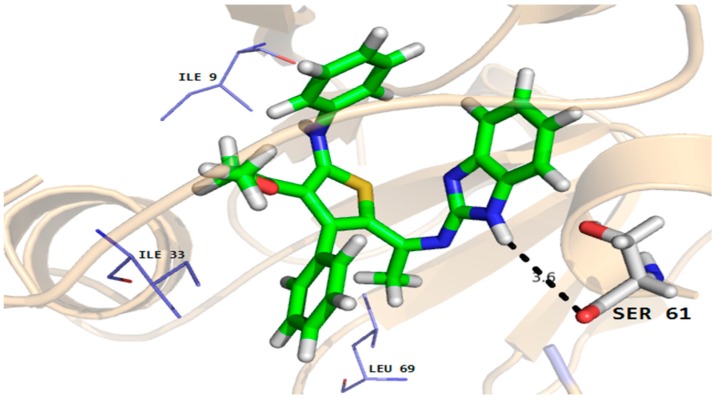
Molecular docking conformation of compound **7** in the active site of *C. albicans* dihydrofolate reductase protein (PDB ID 4HOF) showing hydrogen bonding, hydrophobic and van der Waals interactions.

**Figure 8:**
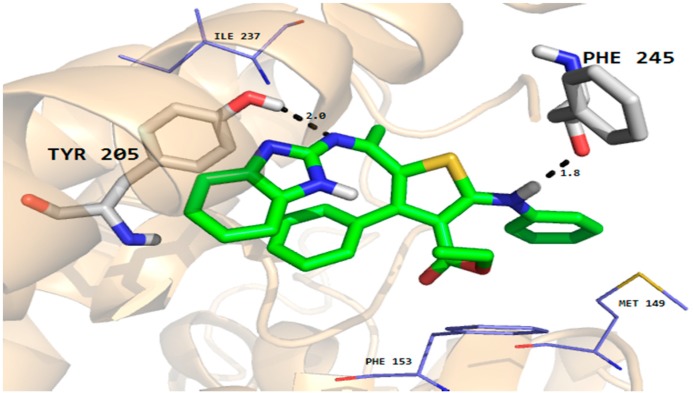
Molecular docking conformation of compound **7** in the active site of rhomboid protease of *E. coli* (PDB ID 3ZMI) showing hydrogen bonding, hydrophobic and van der Waals interactions.

Furthermore, the phenyl ring of the compound formed arene-cation interactions with the active site residue Thr58. Similarly, the docking conformation of compound **5** in the active site of rhomboid protease showed good interactions with the active site residues of this protein. Compound **5** formed three hydrogen bonds with active site residues (Trp157, Met249 and His254) and hydrophobic and VDW interactions with the active site residues (Phe153, Ala239 and Phe245 *etc.*) of rhomboid protease (Figure 6). Compound **5** also interacted with active site residue Tyr 205 via arene-arene interactions. This strong hydrogen bonding and hydrophobic interactions with the active site residues of dihydrofolate reductase and rhomboid protease might be one of the reasons for good antifungal and antibacterial activities shown by this compound in the series. Like compound **5**, good interactions for compounds **8a**, **8b** and **9** were observed with the active site residues of dihydrofolate reductase. With compounds **7** and **10** poor interactions were observed with the active site residues of dihydrofolate reductase. For example, as shown in Figure 7 only one hydrogen bond was established between compound **7** and the active site residues of the target protein. The poor interactions and comparatively low antifungal activities of compounds **7** and **10** might be due to the presence of a more bulky moiety attached to the thiophene ring as compare to compound **5**. Similarly, poor interactions of compounds **7**, **8a**, and **10** with the active site residues of rhomboid protease were observed. As shown in Figure 8, compound **7** which showed less anti-bacterial activity in the series established two hydrogen bonds and few hydrophobic interactions as compared to compound **5** (Figure 6). Overall the docking results showed that the bulky moieties attached to the thiophene ring of compounds **7**, **8a** and **10** might be one of the reasons for their poor interactions and low antifungal and antibacterial activities as compared to compound **5**.

The docking results are unable to explain the good antifungal activity of compound **8b** with a similar bulky group attached to the thiophene ring of the compound as **8a**. On the other hand, we have noted the crucial role and the impact of the electronic effect of chlorine atom of the 4-Cl-phenyl group of **8b**, displaying an electro-donor mesomeric effect (+M), on the antifungal activity (10.9 < % inhibition < 21.5) and (12.3 < % inhibition < 22.8 ) for compounds **8b** and **8a** respectively (Table 2).

**Figure 9:**
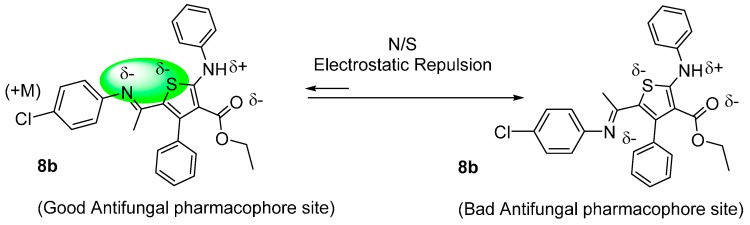
Impact of electronic positive mesomeric effect on geometry and antifungal activity of molecule **8b**.

This means that any supplementary negative surcharge on the nitrogen atom will not beneficial for a perfect *cis* geometric structure of the antifungal pharmacophore site. This is the most notable result of this work, see Figure 9.

### 2.4. POM Analyses

One of the practical problems associated with clinical drugs is the existence of various side effects. For a molecule to be potential drug, besides having a good biological activity, it must have good pharmacokinetic properties in human biological systems. To access the pharmacokinetic profile of the tested compounds, we employed well established *in silico* tools such as Osiris, Petra, and Molinspiration, validated with about 7000 drug molecules available in databases [33].

#### 2.4.1. Osiris Calculations

Structure-based design is now a fairly routine procedure and many potential drugs do not qualify for clinic trials because of ADME-Tox liabilities. One very important class of enzymes, responsible for many ADMET problems, are the P450 cytochromes. Inhibition of these or production of unwanted metabolites can result in many adverse drug reactions. Of the most important program, Osiris is already available online [33] and very useful for its design/prediction of various activities. In our recent publications [34,35,36,37,38,39,40,41,42,43] on the drug design of various pharmacophore sites by using spiro-heterocyclic structures, we have predicted activity and/or inhibition with increasing success in two targets, *Mycobacterium tuberculosis* and HIV. This is done by using a combined electronic/structure docking procedure. The remarkable mutagenicity of divers synthetic molecules classified in the CELERON (Allschwil, Switzerland) database [33], can be used to quantify the role of various organic groups in promoting or interfering the way a drug can associate with DNA [33].

The OSIRIS Property Explorer data shown on this page is an integral part of Actelion’s in-house substance registration system. It allows drawing chemical structures and also calculates various drug-relevant properties whenever a structure is valid. Prediction results are color coded in which the red color shows high risks with undesired effects like mutagenicity or a poor intestinal absorption and a green color indicates drug-conforming behaviour (Table 3).

**Figure 10:**
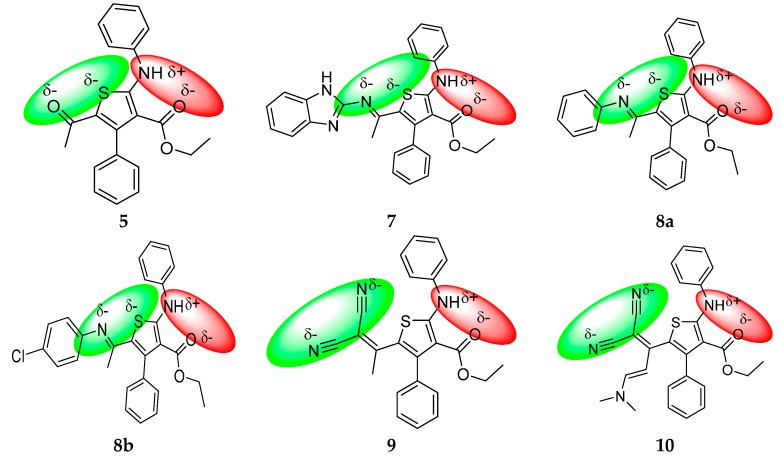
Repartition of partial π-charges of N, O and S atoms of compounds **5**–**10**. The possible antibacterial and antifungal pharmacophores are marked in red and blue color, respectively.

Although two combined antibacterial and antifungal pharmacophore sites coexist in these molecules (Figure 10), the selected compounds of series **5**–**10** showed low to moderate drug scores (DS < 0.50) as compared with the standard drugs used, confirmed by Osiris (Table 3) and Molinspiration (Table 4) calculations. This low bioactivity is due to low hydrosolubility which has a direct impact on bioavailability. In fact most of tested compounds suffer of high cLog*P* (cLog*P* must <5) as it was described by the Lipinski “rule of five”).

#### 2.4.2. Molinspiration Calculations

cLog*P* (octanol/water partition coefficient) is calculated by the methodology developed by Molinspiration and Osiris as a sum of fragment-based contributions and correction factors [44]. The method is very robust and is able to process practically all organic- and organometallic-based molecules. Molecular Polar Surface Area Total Polar Surface Area (TPSA) is calculated by the methodology published by Ertl *et al.* [44] as a sum of fragment contributions. *O*- and *N*-centered polar fragments are considered. PSA has been shown to be a very good descriptor characterizing drug absorption, including intestinal absorption, bioavailability, Caco-2 permeability and blood-brain barrier penetration. Prediction results of the tested compounds of series **5**–**10** with molecular properties (TPSA, GPCR ligand and Ion Channel Modulation (ICM)) are recorded in Table 4. Most of tested compounds have one NH---O or N---HO interactions, too low in comparison with number of NH/O of standard drugs (SD1 and SD2). This explains why most of compounds represent low bioavailability. Drug likeness of series **5**–**10** seems correct and in same range of standard drugs (negative values).

**Figure 11:**
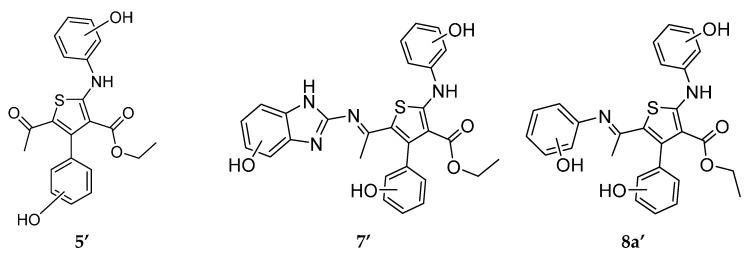

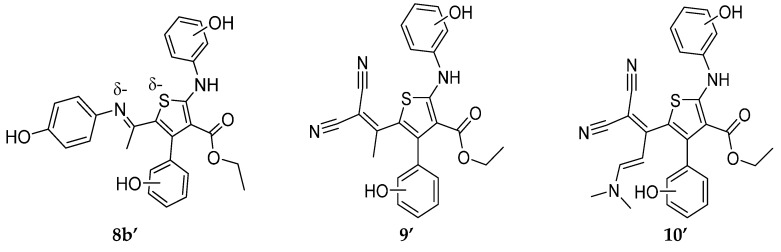
Prospective molecules.

As a result of this information, some prospective leads are under construction in our laboratories (Figure 11). We must avoid the problem of solubility by incorporating more hydroxyl groups on various aryl and heterocyclic groups.

## 3. Experimental Section

### 3.1. Chemistry

#### 3.1.1. General Information

All the chemicals were purchased from various suppliers, including Sigma–Aldrich (St. Louis, MO, USA) and were used without further purification, unless otherwise stated. Melting points were measured on a Gallenkamp melting point apparatus in open glass capillaries and are uncorrected IR Spectra were measured using KBr disc technique at spectral laboratories in King Saud University 6700 FT-IR spectrophotometer. The ^1^H-NMR (400 MHz), and ^13^C-NMR (100 MHz) spectra were recorded on a Jeol-400 NMR spectrometer in deuterated chloroform (CDCl_3_) or dimethyl sulphoxide (DMSO-*d*_6_). Chemical shifts are referred in terms of ppm and *J*-coupling constants are given in Hz. Mass spectra were recorded on a Jeol JMS-600 H machine. Elemental analysis was carried out on Perkin Elmer 2400 Elemental Analyzer; CHN mode. The biological evaluations of the products were carried out in the Medical Mycology Laboratory of the Regional Center for Mycology and Biotechnology of Al-Azhar University, Cairo, Egypt.

#### 3.1.2. Synthetic Procedures

*Ethyl 5-Acetyl-4-phenyl-2-(phenylamino)thiophene-3-carboxylate* (**5**)

To a stirred solution of anhydrous potassium carbonate (10 mmol) in DMF (30 mL) was added ethylbenzoyl acetate (**1**, 10 mmol). After stirring for 1 h, phenyl isothiocyanate (10 mmol) was added to the resulting mixture. Stirring was continued for 2 h, and then chloroacetone (10 mmol) was added portionwise over a period of 30 min. After the addition was complete, the reaction mixture was stirred for an additional 12 h. The reaction was quenched with water (100 mL), and the solid product formed was filtered off, washed with EtOH and dried, Recrystallization from EtOH afforded compound **5** as a pale yellow powder (95%), mp 151–153 °C; IR (KBr) ν_max_ 3229 (NH), 1656 (C=O), 1623 (C=O) cm^−1^; ^1^H-NMR (CDCl_3_) δ 10.69 (s, 1H, D_2_O exchangeable, NH), 7.10–7.40 (m, 10H, ArH), 3.84 (q, 2H, CH_2_, *J* = 7.1 Hz), 1.67 (s, 3H, CH_3_CO), 0.66 (t, 3H, CH_3_, *J* = 7.1Hz); ^13^C-NMR (CDCl_3_) δ 190.55 (CO-CH_3_), 165.54 (COO), 162.67, 146.16, 146.03, 138.67, 136.57, 128.49, 127.40, 126.99, 126.11, 123.49, 118.93, 108.99 (C-Ar), 58.71 (O-CH_2_-CH_3_), 27.51 (CO-CH_3_), 12.00 (O-CH_2_-CH_3_), MS *m*/*z* (%) 367 (2.9%), 366 (9.9%), 365 (M^+^, 38.7%), 114 (99.9%). Anal. Calcd for C_21_H_19_NO_3_S (365.45): C, 69.02; H, 5.24; N, 3.83. Found: 69.13; H, 5.35; N, 3.77%.

##### General Procedure for the Condensation of Thiophene Derivative **5** with Heterocyclic or Aromatic Amines

To a solution of compound **5** (0.365 g, 1 mmol) and 5-amino-1,2,4-triazole, 2-amino-benzimidazole, aniline or 4-chloroaniline (1 mmol) in in absolute ethanol (10 mL) was added ZnCl_2_ (0.1 g) and the reaction mixture was refluxed for 6–7 h. The solvent was evaporated under reduced pressure and the residue was triturated with EtOH, filtered off, washed with EtOH and finally purified by recrystallization from EtOH to give the corresponding condensation products **6**–**8**.

*Ethyl 5-(1-(1H-1,2,4-Triazol-5-ylimino)ethyl)-4-phenyl-2-(phenylamino) thiophene-3-carboxylate* (**6**). White powder (45%), mp < 300 °C; IR (KBr) ν_max_: 3466 (NH), 3233 (NH), 1656 (C=O), 1590 (C=N) cm^−1^; ^1^H-NMR (DMSO-*d*_6_) δ: 10.71 (s, 1H, D_2_O exchangeable, NH), 8.11 (s, 1H, D_2_O exchangeable, NH), 7.38–7.14 (m, 11H, ArH), 3.88 (q, 2H, OCH_2_-CH_3_, *J* = 7.1 Hz,), 2.50 (s, 3H, N=C-CH_3_), 0.71 (t, 3H, OCH_2_-CH_3_, *J* = 7.1 Hz,); MS *m*/*z* (%) 434 (9.1%), 433 (27.8%), 431 (M^+^, 99.9%). Anal. Calcd for C_23_H_21_N_5_O_2_S (431.51): C, 64.02; H, 4.91; N, 16.23. Found: 64.11; H, 4.86; N, 16.28%.

*Ethyl 5-(1-(1H-Benzimidazol-2-ylimino)ethyl)-4-phenyl-2-(phenylamino)thiophene-3*-*carboxylate* (**7**). Yellow powder (56%), mp < 300 °C; IR (KBr) ν_max_: 3427 (NH), 1655 (C=O), 1624 (C=N) cm^−1^; ^1^H-NMR (DMSO-*d*_6_) δ 10.71 (s, 1H, D_2_O exchangeable, NH), 8.03 (s, 1H, D_2_O exchangeable, NH), 7.43–7.13 (m, 14H, ArH), 3.89 (q, 2H, OCH_2_-CH_3_, *J* = 7.2 Hz,), 1.72 (s, 3H, CH_3_-C=N), 0.71 (t, 3H, OCH_2_-CH_3_, *J* = 7.2 Hz); MS *m*/*z* (%) 480 (M^+^, 4.9%). Anal. Calcd for C_28_H_24_N_4_O_2_S (480.58): C, 69.98; H, 5.03; N, 11.66. Found: C, 69.87; H, 5.12; N, 11.56%.

*Ethyl 4-Phenyl-2-(phenylamino)-5-(1-(phenylimino)ethyl)thiophene-3-carboxylate* (**8a**). Green powder (90%), mp 160–162 °C; IR (KBr) ν_max_: 3418 (NH), 1655 (C=O), 1604 (C=N) cm^−1^; ^1^H-NMR (CDCl_3_) δ 10.71 (s, 1H, D_2_O exchangeable, NH), 7.44–6.68 (m, 15H, ArH), 3.89 (q, 2H, OCH_2_-CH_3_, *J* = 7.3 Hz,), 1.71 (s, 3H, CH_3_-C=N), 0.72 (t, 3H, OCH_2_-CH_3_, *J* = 7.3 Hz); MS *m*/*z* (%) 442 (2.7%), 441 (8.5%), 440 (M^+^, 5.5%). Anal. Calcd for C_27_H_24_N_2_O_2_S (440.56): C, 73.61; H, 5.49; N, 6.36. Found: C, 73.72; H, 5.38; N, 6.44%.

*Ethyl 5-(1-(4-Chlorophenylimino)ethyl)-4-phenyl-2-(phenylamino)thiophene-3-carboxylate* (**8b**). Yellowish white powder (98%) , mp 169–171 °C; IR (KBr) ν_max_ 3468 (NH), 1654 (C=O), 1625 (C=N) cm^−1^; ^1^H-NMR (CDCl_3_) δ 10.72 (s, 1H, D_2_O exchangeable, NH), 7.43–7.03 (m, 10H, ArH), 7.33 (d, 2H, *J* = 6 Hz), 7.23 (d, 2H, *J* = 6 Hz), 3.88 (q, 2H, OCH_2_-CH_3_, *J* = 7.1 Hz,), 1.71 (s, 3H, CH_3_), 0.72 (t, 3H, OCH_2_-CH_3_, *J* = 7.1 Hz,). Anal. Calcd for C_27_H_23_N_2_O_2_SCl (475.0): C, 68.27; H, 4.88; N, 5.90. Found: C, 68.33; H, 4.76; N, 5.79

*Ethyl 5-(1,1-Dicyanoprop-1-en-2-yl)-4-phenyl-2-(phenylamino)thiophene-3-carboxylate* (**9**)

To solution of thiophene **5** (0.365 g, 1 mmol) and malononitrile (0.066 g, 1 mmol) in ethanol (20 mL) few drops of piperidine were added and the reaction mixture was refluxed for 5 h. The resulting solid product was collected by filtration, dried and recrystallized from ethanol to afford a purple powder in 87% yield, mp 159–161 °C; IR (KBr) ν_max_ 3466 (NH), 2366 (C≡N), 1656 (C=O) cm^−1^; ^1^H-NMR (CDCl_3_) δ 10.71 (s, 1H, D_2_O exchangeable, NH), 7.40–7.38 (m, 10H, ArH), 3.89 (q, 2 H, OCH_2_-CH_3_, J = 7.0 Hz), 2.31 (s, 3H, CH_3_), 0.72 (t, 3H, OCH_2_-CH_3_, J = 7.0 Hz). Anal. Calcd for C_24_H_19_N_3_O_2_S (413.49): C, 69.71; H, 4.63; N, 10.16. Found: 69.65; H, 4.56; N, 10.22%.

*Ethyl 5-(1,1-Dicyano-4-(dimethylamino)buta-1,3-dien-2-yl)-4-phenyl-2-(phenylamino)thiophene-3-carboxylate* (**10**)

A mixture of compound **9** (1.5 g, 5 mmol) and DMF-DMA (2 mL) was heated under reflux for 8 h, then left to cool to room temperature. The dark yellow precipitate was filtered off, washed with petroleum ether, and dried. Re-crystallization from EtOH to afford compound **10**. Yellow powder (82%) yield, mp. 168–170 °C; IR (KBr) ν_max_ 3451 (NH), 2370 (C≡N), 2340 (C≡N), 1666 (C=O) cm^−1^; ^1^H-NMR (CDCl_3_) δ 10.71 (s, 1H, D_2_O exchangeable, NH), 7.43–7.14 (m, 10H, ArH), 6.9 (d, 1H, J = 12.1 Hz, N-CH=CH), 5.3 (d, 1H, J = 12.1 Hz, N-CH=CH), 3.89 (q, 2 H, OCH_2_-CH_3_, J = 7.2 Hz), 3.33 (s, 3H, CH_3_-N), 3.19 (s, 3H, CH_3_-N), 0.72 (t, 3H, OCH_2_-CH_3_, J = 7.2 Hz,); MS m/z (%) 468 (M^+^, 8.8%), 134 (99.9%), 107 (91%), 55 (89.3%), 83 (96%). Anal. Calcd for C_27_H_24_N_4_O_2_S (468.57): C, 69.21; H, 5.16; N, 11.96. Found: 69.33; H, 5.25; N, 11.88%.

### 3.2. Antimicrobial Activity

#### 3.2.1. Antibacterial Activity

*In vitro* antibacterial screening tests of the synthesized compound was carried out against four bacterial strains: two Gram-positive (*Streptococcus pneumonia* and *Bacillis subtilis*) and two Gram-negative (*Pseudomonas aeruginosa* and *Escherichia coli*). The disc diffusion method [45,46] was used in this assay and each experiment was performed in triplicate. Readings of the zone of inhibition, which are shown in Table 1, represent the mean value of three readings. Ampicillin and gentamicin were used as standard drugs in this assay. The data obtained show that the compounds have good antibacterial activity. The antibacterial activity and inhibition zone around the tested compound can be caused by their bactericide effects (killing the bacteria) or by their bacteriostatic effects (inhibiting multiplication of bacteria by blocking their active sites on surface or inside bacterial cell).

#### 3.2.2. Antifungal Activity

The synthesized thiophene derivatives **5**, **7**, **8a**–**b**, **9** and **10**, were subjected to *in vitro* antifungal activity against four fungal strains (*Aspergillus fumigates, Syncephalastrum racemosum, Geotricum candidum* and *Candida albicans*) using the disk dilution method [47]. The results are shown in Table 2. Amphotericin B was used as a standard drug. Percentage inhibition value of biological active samples was evaluated by following formula:

PIFG = [GDTC/GDC] × 100

PIFG: Percent inhibition of fungal growth. GDTC: Growth diameter in test compound (mm). GDC: Growt diameter in control (mm).

It is concluded from the antibacterial and antifungal activities that the compounds show good antibacterial and antifungal activities so they may be used as potential antimicrobial drug leads.

## 4. Conclusions

Armed derivatives of thiophene were synthesized in moderate to excellent yield of **6**–**7** is not “excellent” using ethyl 5-acetyl-4-phenyl-2-(phenyl-amino)thiophene-3-carboxylate (**5**) as principal precursor. The tested compounds, thiophene analogs armed at different positions namely at C3, C5 and substituted at C2 and C3 in the thiophene subunit were evaluated for their *in vitro* antimicrobial activity against selected pathogenic bacteria and fungus. The preliminary structure-activity relationship (SAR) analysis suggested that the introduction of appropriate substituent groups at position **5** of the thiophene ring enhanced the antibacterial activities of these compounds. Most of the newly synthesized products have promising antimicrobial activity, comparable to the first line standard drugs. Although we did our best to build the most efficient bioactive leads, extensive synthesis in the analogue series of compounds **5**–**10** is still needed in order to revise some of the structural shortcomings of the pharmacophoric sites and to produce more efficient drugs on the basis of a POM-guided better understanding of the relationship between the physicochemical properties and biological activity observed for these compounds.

## Figures and Tables

**Table 1:** Antibacterial activity of the synthesized thiophene derivatives **5**, **7**, **8a**–**b**, **9** and **10**.

Compound	Gram Positive Bacteria		Gram Negative Bacteria	
	*Streptococcus pneumoniae*	*Bacillis subtilis*	*Pseudomonas aeruginosa*	*Escherichia coli*
**5**	13.7 ± 0.31	10.5 ± 0.32	11.7 ± 0.41	12.5 ± 0.48
**7**	18.3 ± 0.25	22.6 ± 0.44	19.3 ± 0.52	17.8 ± 0.44
**8a**	19.5 ± 0.44	29.8 ± 0.58	12.3 ± 0.25	17.6 ± 0.19
**8b**	17.8 ± 0.58	20.1 ± 0.39	10.9 ± 0.31	15.3 ± 0.32
**9**	14.1 ± 0.52	16.3 ± 0.48	11.1 ± 0.42	11.4 ± 0.37
**10**	13.1 ± 0.43	10.8 ± 0.39	13.4 ± 0.34	12.3 ± 0.47
Ampicillin	23.8 ± 0.20	32.4 ± 0.30	-	-
Gentamicin	-	-	17.3 ± 0.11	19.9 ± 0.30

Data are expressed as mean ± SD.

**Table 2:** Antifungal activity of the synthesized thiophene derivatives **5**, **7**, **8a**–**b**, **9** and **10**.

**Compound**	Fungal Strains ^[a]^			
	*Aspergillus fumigates*	*Syncephalastrum racemosum*	*Geotricum candidum*	*Candida albicans*
**5**	13.8 ± 0.42	14.1 ± 0.35	13.2 ± 0.34	10.8 ± 0.22
**7**	20.6 ± 0.58	16.7 ± 0.33	22.4 ± 0.36	17.6 ± 0.58
**8a**	22.3 ± 0.25	16.5 ± 0.25	25.8 ± 0.58	12.3 ± 0.35
**8b**	20.6 ± 0.35	14.8 ± 0.34	21.5 ± 0.62	10.9 ± 0.18
**9**	14.8 ± 0.42	15.3 ± 0.51	14.8 ± 0.46	11.9 ± 0.38
**10**	19.8 ± 0.63	16.8 ± 0.46	13.9 ± 0.48	14.6 ± 0.50
**SD** ^[b]^	23.7 ± 0.1	19.7 ± 0.2	28.7 ± 0.2	25.4 ± 0.1

^[a]^ Data are expressed as mean ± SD; ^[b]^ SD: Standard Drug (amphotericin B).

**Table 3:** Osiris calculations of compounds **5**, **8a**, **8b**, **9**, **l0** and standard drugs **SD1**, **2**.

Compound	MW	Toxicity Risks ^[a]^				Drug-Score ^[b]^			
		MUT	TUMO	IRRI	REP	CLP	S	DL	DS
**5**	365	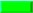	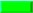	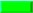	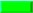	5.10	−6.56	−2.10	0.22
**7**	480	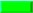	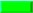	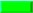	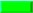	6.51	−8.26	−0.96	0.15
**8a**	440	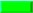	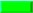	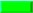	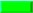	6.54	−7.78	−2.01	0.15
**8b**	474	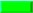	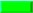	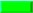	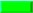	7.14	−8.52	−2.94	0.12
**9**	413	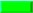	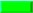	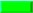	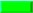	5.63	−6.94	−10.61	0.16
**10**	468	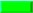	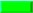	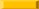	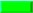	5.13	−6.56	−9.78	0.14
**SD1** ^[c]^	349	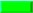	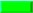	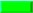	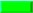	−0.04	−1.57	10.72	0.91
**SD2** ^[c]^	477	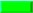	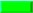	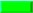	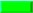	−4.03	−1.18	4.88	0.77

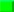
: not toxic, 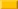: slightly toxic; ^[a]^ MUT: mutagenic, TUMO: tumorigenic, IRRI: irritant, REP: reproductive effective; ^[b]^ CLP: cLog*P*, S: Solubility, DL: Druglikness, DS: Drug-Score; ^[c]^ Standard drugs (**SD1**: ampicillin, **SD2**: gentamicin).

**Table 4:** Molinspiration calculations of compounds **5**, **8a**, **8b**, **9**, **l0** and standard drugs **SD1**,**2**.

Compound	Physico-Chemical Properties ^[a]^					Drug Likeness ^[b]^					
	TPSA	ONH	VIOL	ROTB	VOL	GPC	ICM	KI	NRL	PI	EI
**5**	55	1	1	7	327	−0.48	−0.53	−0.38	−0.54	−0.76	−0.46
**7**	75	1	1	9	434	−0.47	−0.46	−0.35	−0.54	−0.68	−0.29
**8a**	51	1	1	8	403	−0.42	−0.51	−0.30	−0.40	−0.69	−0.33
**8b**	51	1	1	8	416	−0.41	−0.50	−0.30	−0.41	−0.70	−0.35
**9**	86	1	1	7	369	−0.48	−0.50	−0.26	−0.36	−0.58	−0.34
**10**	89	1	1	9	426	−0.43	−0.48	−0.25	−0.46	−0.56	−0.34
**SD1** ^[c]^	113	4	0	-	299	−0.56	−0.55	−0.90	−0.87	NC	NC
**SD2** ^[c]^	206	12	2	-	454	−0.46	−0.24	−0.76	−1.05	NC	NC

^[a]^ TPSA: Total polar surface area, ONH: O---HN or OH---N interaction, VIOL: number of violations, VOL: volume; ^[b]^ ICM: Ion channel modulator, KI: Kinase inhibitor, NRL: Nuclear receptor ligand, PI: Protease inhibitor, EI: Enzyme inhibitor. NC: not calculated; ^[c]^ Standard drugs (**SD1**: ampicillin, **SD2**: gentamicin).

## References

[1] Bohlman F., Zdero C., Gronowitz S. (1986). Thiophene and Its Derivatives.

[2] Perry N.B., Blunt J.W., Munro M.H.G. (1988). Cytotoxic pigments from New Zealand sponges of the genus latrunculia: Discorhabdins A, B and C. Tetrahedron.

[3] Kobayashi J., Cheng J.-F., Ishibashi M., Nakamura H., Ohizumi Y., Hirata Y., Sasaki T., Lu H., Clardy J., Prianosin A. (1987). A novel antileukemic alkaloid from the Okinawan marine sponge Prianosmelanos. Tetrahedron Lett..

[4] Press J.B., Gronowitz S. (1991). Thiophene and its derivatives. The Chemistry of Heterocyclic Compounds, Part 4.

[5] Xiaoyun L., Baojie W., Scott G.F., Qidong Y. (2011). Design, synthesis and anti-tubercular evaluation of new 2-acylated and 2-alkylated amino-5-(4-(benzyloxy)phenyl)thiophene-3-carboxylic acid derivatives, Part 1. Eur. J. Med. Chem..

[6] Hartman G.D., Ansdale P.A. (1989). Substituted Thieno[2,3-*b*]thiophene-2-sulfonamides as Antiglaucoma Agents. U.S. Patent.

[7] Hafez H.N., El-Gazzar A.B.A. (2008). Design and synthesis of 3-pyrazolyl-thiophene, thieno[2,3-*d*]pyrimidines as new bioactive and pharmacological activities. Bioorg. Med. Chem. Lett..

[8] Fiesselmann H., Li J.J. (2006). Thiophene synthesis. Name Reactions.

[9] Gabar H.M., Bagley M.C. (2011). Regioselective synthesis and biological evaluation of some novel thiophene containing heterocyclic scaffolds as potential chemotherapeutic agents. Eur. J. Chem..

[10] Josué S.B.F., Erika M.D.C., Joel J.J., Flavia M.D.S. (2011). A new protocol for the synthesis of 2-aminothiophnes through the Gewald reaction in solvent-free conditions. Heterocycl. Lett..

[11] Sommen G., Comel A., Kirsch G. (2003). Preparation of thieno[2,3-*b*]pyrroles starting from ketene-N,S-acetals. Tetrahedron.

[12] David T., Enrico P., Dorothe’e H., Zhanjie X., Serge S., Stéphanie H., Gilbert K., Pierre S. (2009). One-pot synthesis of new tetrasubstitutedthiophenes and selenophenes. Tetrahedron.

[13] Bayrak H., Demirbas A., Demirbas N., Karaoglu S. (2009). Synthesis of some new 1,2,4-triazoles starting from isonicotinic acid hydrazide and evaluation of their antimicrobial activities. Eur. J. Med. Chem..

[14] Güzeldemirci N.U., Küçükbasmaci O. (2010). Synthesis and antimicrobial activity evaluation of new 1,2,4-triazoles and 1,3,4-thiadiazoles bearing imidazo[2,1-*b*]thiazole moiety. Eur. J. Med. Chem..

[15] Mikami Y., YazawaUno K.J., Matsumae A. (1990). *In vitro* activity of amphotericin B, flucytosine, fluconazole and miconazole against clinically isolated *Candida albicans*, *Aspergillus funigatus* and *Trichosporon beigelii*. Chemotherapy.

[16] Tozkoparan B., Küpeli E., Yesilada E., Ertan M. (2007). Preparation of 5-aryl-3-alkylthio-l,2,4-triazoles and corresponding sulfones with antiinflammatory–analgesic activity. Bioorg. Med. Chem..

[17] Abdel-Megeed A.M., Abdel-Rahman H.M., Alkaramany G.-E.S., El-Gendy M.A. (2009). Design, synthesis and molecular modeling study of acylated 1,2,4-triazole-3-acetates with potential anti-inflammatory activity. Eur. J. Med. Chem..

[18] Holla B.S., Veerandra B., Shivananda M.K., Poojary B. (2003). Synthesis characterization and anticancer activity studies on some Mannich bases derived from 1,2,4-triazoles. Eur. J. Med. Chem..

[19] Deepika S., Balasubramanian N., Pradeep K., Abraham J. (2009). Synthesis and QSAR evaluation of 2-(substituted phenyl)-1*H*-benzimidazoles and [2-(substituted phenyl)-benzimidazol-1-yl]-pyridin-3-yl-methanones. Eur. J. Med. Chem..

[20] Tewari A.K., Mishra A. (2006). Synthesis and antiviral activities of *N*-substituted-2-substituted-benzimidazole derivatives. Indian J. Chem..

[21] Demirayak S., Mohsen U.A., Karaburun A.C. (2002). Synthesis and anticancer and anti-HIV testing of some pyrazino [1,2-*a*]benzimidazole derivatives. Eur. J. Med. Chem..

[22] Penning T.D., Zhu G., Gandhi V.V., Gong J., Thomas S., Lubisch W. (2008). Discovery and SAR of 2-(1-propylpiperidin-4-yl)-1*H*-benzimidazole-4-carboxamide: A potent inhibitor of poly (ADP-ribose) polymerase (PARP) for the treatment of cancer. Bioorg. Med. Chem..

[23] Kus C., Atlanlar N. (2003). Synthesis of some new benzimidazolecarbamate derivatives for evaluation of antifungal activity. Turk. J. Chem..

[24] Ayhan-Kilcigil G., Kus C., Ozdamar E.D., Can-Eke B., Iscan M. (2007). Synthesis and antioxidant capacities of some new benzimidazole derivatives. Arch. Pharm. Chem. Life Sci..

[25] Mabkhot Y.N., Kheder N.A., Al-Majid A.M. (2010). Facile and convenient synthesis of newthieno[2,3-*b*]thiophene derivatives. Molecules.

[26] Mabkhoot Y.N. (2009). Synthesis and analysis of some bis-heterocyclic compounds containing sulphur. Molecules.

[27] Mabkhoot Y.N., Al-Majid A.M., Barakat A., Alshahrani S., Siddiqui Y. (2011). 1,1’-(3-Methyl-4-phenylthieno[2,3-*b*]thiophene-2,5-diyl)diethanone as a building block in heterocyclic synthesis. novel synthesis of some pyrazole and pyrimidine derivatives. Molecules.

[28] Mabkhoot Y.N., Kheder N.A., Farag A.M. (2013). Synthesis and antimicrobial activity of some newthieno[2,3-*b*]thiophene derivatives. Molecules.

[29] Vaara M. (1993). Antibiotic-supersusceptible mutants of Escherichia coli and Salmonella typhimurium. Antimicrob. Agents Chemother..

[30] Nikaido H., Vaara M. (1985). Molecular basis of bacterial outer membrane permeability. Microbiol. Rev..

[31] Plésiat P., Nikaido H. (1992). Outer membranes of gram-negative bacteria are permeable to steroid probes. Mol. Microbiol..

[32] Chemical Computing Group (2013). Molecular Operating Environment (MOE).

[33] Osiris Software. http://www.organic-chemistry.org/prog/peo/.

[34] Waring M.J., Ben-Hadda T., Kotchevar A.T., Ramdani A., Touzani R., Elkadiri S., Hakkou A., Bouakka M., Ellis T. (2002). 2,3-Bifunctionalized Quinoxalines: Synthesis, DNA Interactions and Evaluation of Anticancer, Anti-tuberculosis and Antifungal Activity. Molecules.

[35] Ben Hadda T., Benchat N., El-Bali B., Abouricha S., Moueqqit M., Mimouni M. (2003). Impact of Dimroth Rearrangement on anti-Tuberculosis Activity of 3-armed-Imidazo[1.2-*a*]pyrimidines IMP (-Pyridines) IP. Med. Pharm. Chem..

[36] Anaflous A., Benchat N., Mimouni M., Abouricha S., Ben Hadda T., El-Bali B., Hakkou A., Hacht B. (2004). Armed Imidazo[1,2-*a*]pyrimidines ((Pyridines): Evaluation of Antibacterial Activity. Lett. Drug Des. Discov..

[37] Ben Hadda T., Rahima B., Kerbal A., Baba B.F., Akkurt M., Demailly G., Benazza M. (2007). Synthesis and antitubercular activity of spiroheterocycles: 2,2′,4′,5′-tetra-substituted-1,2,2′,4′-tetrahydro-4*H*-spiro[isoquinoline-3,3′-pyrazol]-4-ones. Arkivoc.

[38] Ben Hadda T., Rahima B., Kerbal A., Baba B.F., Akkurt M., Demailly G., Benazza M. (2008). Looking for a new antitubercular pharmacophore site: Synthesis and bioactivity of spiroheterocycles 2,3′,4′-tri-subsituted-1,2-dihydro-4*H*,4′*H*-spiro[isoquinoline-3,5′-isoxazol]-4-ones. Arkivoc.

[39] Houari G.A., Kerbal A., Bennani B., Baba M.F., Daoudi M., Ben Hadda T. (2008). Drug design of new antitubercular agents: 1,3-dipolar cycloaddition reaction of arylnitriloxides and 3-para-methoxy-benzylidene-isochroman-4-ones. Arkivoc.

[40] Ben Hadda T., Genc Z.K., Masand V.H., Nebbache N., Warad I., Jodeh S., Genc M., Mabkhot Y.N., Barakat A., Salgado-Zamora H. (2015). Computational POM and DFT Evaluation of Experimental *in-vitro* Cancer Inhibition of Staurosporine-Ruthenium(II) Complexes: The Power Force of Organometallics in Drug Design. Acta Chim. Slov..

[41] Zunino F., Kotchevar A.T., Waring M., Daoudi M., Larbi N.B., Mimouni M., Sam N., Ben Hadda T. (2002). Novel Cytotoxic Oxopyridoindolizines: iso-Propyl-7,8,9-tri-chloro-6,7,8,9-tetra-hydro-5-oxo-pyrido[2,3-*a*]indolizine-10-carboxylates. Molecules.

[42] Ben Hadda T., Kotchevar A.T., Daoudi M., Bennani B., Larbi N.B., Kerbal A. (2005). Anti-tumour Activity of Some Polydentate N-Ligands: *N*,*N*-bis-(3-Substituted-5-Methylpyrazol-1yl Methyl) Arylamines and *N*,*N*,*N*′,*N*′-Tetra-[(3-Substituted-5-Methylpyrazol-1yl]Para-Phenylene diamines. Lett. Drug Des. Discov..

[43] Bennani B., Kerbal A., Daoudi M., Baba B.F., Houari G.A., Jalbout A.F., Mimouni M., Benazza M., Demailly G., Akkurt M. (2007). Combined drug design of potential Mycobacterium tuberculosis and HIV-1 inhibitors: 3’,4’-di-substituted -4’*H*-spiro[isothiochromene-3,5’-isoxazol]-4(1*H*)-one. Arkivoc.

[44] Ertl P., Rohde B., Selzer P. (2000). Fast Calculation of Molecular Polar Surface Area as a Sum of Fragment-Based Contributions and Its Application to the Prediction of Drug Transport Properties. J. Med. Chem..

[45] Jafri L., Ansari F.L., Jamil M., Kalsoom S., Qureishi S., Mirza B. (2012). Microwave-assisted synthesis and bioevaluation of some semicarbazones. Chem. Biol. Drug Des..

[46] Rehman A., Choudhary M.I., Thomsen W.J. (2001). Bioassay Techniques for Drug Development.

[47] Arikan S., Paetznick V., Rex J.H. (2002). Comparative evaluation of disk diffusion with microdilution assay in susceptibility testing of caspofungin against *Aspergillus* and *Fusarium* isolates. Antimicrob. Agents Chemother..

